# The Bacterium *P. aeruginosa* Disperses Ordered Membrane Domains by Targeting Phase Boundaries

**DOI:** 10.3390/biom15030341

**Published:** 2025-02-27

**Authors:** Kai Stober, Fabian Schwerdtfeger, Sahaja Aigal, Yves Mely, Winfried Römer

**Affiliations:** 1 Faculty of Biology, University of Freiburg, Schänzlestraße 1, 79104 Freiburg, Germany; kai.stober@bioss.uni-freiburg.de (K.S.);; 2 Signalling Research Centres BIOSS and CIBSS, University of Freiburg, Schänzlestraße 18, 79104 Freiburg, Germany; 3 Laboratoire de Bioimagerie et Pathologies, UMR 7021 CNRS, Faculté de Pharmacie, Université de Strasbourg, 67401 Illkirch CEDEX, France

**Keywords:** supported lipid bilayer, membrane dynamics, lipid rafts, *Pseudomonas aeruginosa*, glycosphingolipids, Gb3, bacterial adhesion

## Abstract

Various pathogens use receptors on the host’s plasma membrane for their cellular uptake. For the bacterium *Pseudomonas aeruginosa*, interactions between its lectin LecA and the host cell glycosphingolipid globotriaosylceramide (also known as Gb3) are crucial for its internalization via the so-called lipid zipper mechanism. In this study, we investigated the interactions of the *P. aeruginosa* strain PAO1 with phase-separated lipid bilayers containing Gb3. Surprisingly, bacteria are mostly bound to the interphase of liquid-ordered (Lo) and liquid-disordered (Ld) membrane domains. Simultaneously with the formation of bacterial aggregates and the accumulation of membrane lipids, the lipid bilayers were drastically reorganized and Lo domains were dissolved. Surprisingly, Gb3 was found to play a role in the localization of the bacterium at the interface, less so LecA. When microspheres were used as a minimal mimic of the bacterium, these beads also localized preferentially at the Lo–Ld phase boundaries, but in contrast to living bacteria, beads were unable to cause membrane reorganization and dissolution of the Lo domain, even when coated with LecA. Targeting phase boundaries as “weak points” in membranes and thereby reorganizing and destabilizing the host cell plasma membrane could be an attractive entry strategy for *P. aeruginosa* and many other bacteria and viruses.

## 1. Introduction

Bacterial pathogens have a wide range of virulence factors that facilitate successful infection of host cells. An important mechanism is their ability to interact directly or indirectly with the host’s plasma membrane [1,2]. In this context, it is known that some bacteria deform and remodel the plasma membranes of host cells to promote infection through intracellular invasion or biofilm formation on the cell surface [3,4,5,6,7].

One such peculiar pathogen is *Pseudomonas aeruginosa*, which is classified as critical by the World Health Organization, because of the occurrence of carbapenem-resistant strains [8]. Besides its antibiotic resistance, it has a wide variety of possibilities to promote its virulence. These include biofilm formation, quorum sensing systems, pili, multiple types of secretion systems, and released factors like exotoxins or other enzymes, among others [9,10]. Additionally, *P. aeruginosa* can utilize its carbohydrate-binding proteins, the lectins LecA and LecB (also known as PA-IL and PA-IIL), to interact with α-galactose and fucose/mannose moieties on host cells, respectively. Both LecA and LecB are controlled by quorum sensing and play an important role during adhesion and biofilm formation [11,12,13,14]. The glycosphingolipid globotriaosylceramide (also referred to as Gb3, CD77, and P^K^ blood group antigen) in epithelial host plasma membranes functions as a receptor for the homo-tetrameric LecA, which is partly located on the outer membrane of the bacterium [15,16]. The LecA-induced Gb3 clustering acts as a lipid zipper, which leads to a deformation of the plasma membrane and allows bacteria to invade host cells [6,17]. Membrane deformation is not the only effect that is triggered by the LecA–Gb3 interaction. It also activates host cell signaling by Abl-independent induction of CrkII phosphorylation resulting in further promotion of the bacterial uptake [18]. The signal transmission from the outside, where LecA binds Gb3, to the intracellular space could be caused by the interdigitation of glycosphingolipids with long fatty acyl chains [19]. Brandel et al. observed that LecA–Gb3 interactions induce a specialized plasma membrane domain composed of the GPI-anchored protein CD59 next to saturated Gb3 species at the extracellular membrane leaflet, the phospholipid PIP_3_ at the cytosolic membrane leaflet, and flotillins accumulating from the cytosol, which all promoted the invasion process [20]. It has been established for some time that *P. aeruginosa* has the ability to alter and influence host cell membranes [3]. However, only recently it has been shown that LecA is a virulence factor of *P. aeruginosa* that can remodel membrane domains [21]. By utilizing fluorescence microscopy as well as atomic force microscopy, it was confirmed that soluble LecA can remodel lipid bilayers into multilayers. This was observed with supported lipid bilayers (SLBs), a synthetic model membrane system [21]. These artificial membrane systems can aid in understanding certain aspects of biological processes, as mammalian cells and bacteria as well as their interactions with each other are extremely complex, and therefore difficult to study [22,23]. However, with these synthetic systems, we are capable of establishing controlled environments with fewer parameters, which allows the reconstruction of cellular processes and elucidation of their key mechanisms. Thereby, they can be set up to possess desired properties, which are mainly dependent on the lipid composition of the SLBs. For instance, a ternary lipid mixture containing saturated and unsaturated phospholipids as well as cholesterol will form liquid-ordered (Lo) and liquid-disordered (Ld) domains. This is due to the properties of the saturated phospholipids, which can be packed tightly together with cholesterol, excluding water, and forming a rigid domain, the Lo domain [24,25,26]. It was proven that the naturally occurring tetrameric LecA (as well as an engineered dimeric version of the lectin) can induce the dispersion of Lo domains [21]. Lo domains are frequently considered as models for “raft-like” domains, which are believed to correspond to transient micro- and nanodomains in plasma membranes acting as signaling platforms in mammalian cells [20,27]. These domains are thought to exhibit a high content of Gb3, because of the high saturation of its acyl chains, allowing *P. aeruginosa* to exploit these signaling platforms and invade non-phagocytic cells with the help of its lectin LecA [20]. To counter infections with *P. aeruginosa*, several glycomimetics have been developed to bind LecA and inhibit its ability to interact with its substrate, decreasing bacterial invasiveness into host cells significantly [28,29].

In this paper, we investigated the behavior of the bacterium *P. aeruginosa* during its initial contact with a lipid bilayer. We found that bacteria preferentially localized to the Lo–Ld phase boundaries. Moreover, bacteria were observed to cluster at the SLB surface and induce the dispersion of Lo domains. Furthermore, we looked closer at the role of LecA–Gb3 interactions in this context. With increasing Gb3 concentration, LecA-mediated attachment and clustering of bacteria occurred faster than without LecA, but the LecA–Gb3 interactions contributed rather slightly to the dispersion of the ordered membrane domains, as these were also dissolved when LecA was neutralized and Gb3 was absent in lipid bilayers.

## 2. Materials and Methods

### 2.1. Lipids and Lipid Mixtures

1,2-dioleoyl-sn-glycero-3-phosphocholine (DOPC), porcine brain-sphingomyelin (SM), cholesterol, and FSL-Gb3 were bought from Sigma Aldrich (Darmstadt, Germany), while Gb3 mix was purchased from Cayman Chemical (Ann Arbor, MI, USA), and the fluorescent Texas Red 1,2-dihexadecanoyl-*sn*-glycero-3-phosphoethanolamine (Texas Red-DHPE) was purchased from ThermoFisher Scientific (Darmstadt, Germany). Lipids were dissolved in chloroform, while the glycolipids Gb3 and FSL-Gb3 were stored in a mixture of two parts chloroform and one part methanol. Lipid mixtures had a lipid concentration of 0.25 mg mL^−1^, and compositions were calculated and prepared according to desired molar percentages (mol-%). Stocks and mixtures were overlaid with argon and stored at −20 °C.

### 2.2. Supported Lipid Bilayer Formation

SLBs were prepared as described before [30]. Lipid mixtures were dried under nitrogen flow and fully desiccated in a vacuum. Afterwards, lipids were resuspended in MilliQ water by heating them to 55 °C and extensive vortexing, thus forming multi-lamellar vesicles. The vesicles were extruded (polycarbonate membrane, pore size: 50 nm, Avestin, Ottawa, ON, Canada) 15 times in three cycles with a 15 min incubation step at 55 °C in between each extrusion cycle. Mica (10 mm, V1 quality, Plano GmbH, Wetzlar, Germany) was glued (Norland optical adhesive 61, Norland Products, Jamesburg, NJ, USA) onto 30 mm glass slides (ThermoFisher Scientific, Darmstadt, Germany). Subsequently, chambers were created by gluing 8 × 8 mm glass cylinders (Pyrex^®^, Sigma Aldrich, Darmstadt, Germany) on top of freshly cut mica. Two drops (roughly 50–60 µL total) of extruded vesicles were added to each chamber, as well as 150 µL of 10 mM calcium chloride (Carl Roth GmbH, Carl Roth GmbH, Karlsruhe, Germany) to form SLBs. After 30 min incubation at 55 °C, SLBs were washed with pre-warmed PBS (ThermoFisher Scientific, Darmstadt, Germany) and incubated again at 55 °C for another 30 min. SLBs were cooled down slowly overnight and imaged the next day.

### 2.3. Bacterial Culture

The GFP-expressing *Pseudomonas aeruginosa* PAO1 strain was grown in LB medium (Carl Roth GmbH, Karlsruhe, Germany) containing 50 µg mL^−1^ gentamicin (Sigma-Aldrich, Darmstadt, Germany), shaking at 650 rpm and 37 °C for 16 h. The next day, bacteria were centrifuged for 12 min at 3000× *g* and resuspended in PBS. After the determination of the absorbance at 600 nm, bacteria were diluted with PBS and added to the SLB. From the addition of the bacteria onto the SLB to the start of the imaging roughly 5 min passed. Therefore, the time series starts with the 5 min time point. The LecA knockout mutant [6] was handled the same way. To block LecA–Gb3 interactions, 10 mM *para*-nitrophenyl-α-D-galactopyranosid (PNPG, Sigma Aldrich, Darmstadt, Germany) was utilized.

### 2.4. LecA Production, Biotinylation, and LecA Bead Preparation

LecA was produced using *Escherichia coli* BL21 (DE3) carrying the plasmid pET25pa1l as previously described [15]. To couple LecA to beads, 1.5 mg mL^−1^ LecA was biotinylated by adding 10 mM NHS-PEG_4_-biotin (ThermoFisher Scientific, Darmstadt, Germany) and incubating for 2 h. Unbound NHS-PEG_4_-biotin was removed through dialysis (10K MWCO, ThermoFisher Scientific, Darmstadt, Germany) in 5 L MilliQ water for 1 h at room temperature. This process was repeated once, followed by overnight dialysis at 4 °C in PBS. Biotinylation was confirmed with a HABA assay using a Pierce Biotin Quantification Kit (ThermoFisher Scientific, Darmstadt, Germany).

LecA-coupled beads were prepared as previously reported [31,32]. Streptavidin-coated microspheres (0.99 µm, FlashRed, Bangs Laboratories, Fishers, IN, USA) were washed three times with PBS, centrifuged for 5 min at 5000× *g*, and the supernatant was removed. The pelleted microspheres were resuspended in PBS, and biotinylated LecA was added accordingly to the loading capacity of the beads. After 30 min of incubation at room temperature while shaking, the beads with coupled LecA were washed thrice with PBS and stored at 4 °C. For each experiment, the number of beads was adjusted to 3 × 10^8^.

### 2.5. Microscopy

Experiments with SLBs and *P. aeruginosa* were performed with a Nikon A1R laser scanning confocal microscope using a 60x oil immersion objective (numerical aperture 1.49) and 488 nm and 561 nm laser lines. Experiments involving LecA-coated beads on SLBs were either performed on the same microscope, or on a Nikon TIRF microscope with a 100× oil immersion objective (numerical aperture 1.49), and laser lines at 561 nm and 647 nm.

### 2.6. Image Analysis

Captured images and image series from fluorescence microscopy experiments were analyzed by custom ImageJ (v1.54, NIH, Bethesda, MD, USA) scripts that are available on GitHub: https://github.com/taras-sych/SLB-tools (accessed on 2 December 2022). Both differentiating bacteria from the background and defining Lo and Ld domains were accomplished by utilization of thresholds. Interfaces between Lo and Ld domains are assigned by a 1 µm wide line between phases, and bacteria in each location (Lo, Ld, Lo–Ld interface) were counted. The areas of Lo and Ld phases were calculated as a percentage of the total area of the frame. Bacterial clustering was quantified as follows: An intensity value was determined to separate single bacteria from noise. This value was used as a threshold to differentiate bacterial clusters from the background, which allowed the calculation of cluster areas. Statistical analysis was performed using the Mann–Whitney U test. In box plots, the box indicates the interquartile range (25–75% data points), with whiskers and outliers determined using the Tukey method. The horizontal line indicates the median and the “+” symbol the mean value.

The bacterial localization percentage was calculated as the bacterial density [PAO1·μm^−2^] (per normalized interface or domain area) divided by the sum of all domains and multiplied by 100. Normalized bacterial density values were averaged over three consecutive time points to achieve more robust data. Unpaired t-tests and Wilcoxon matched-pairs signed rank tests were used for statistical analysis. The bacterial localization speed was calculated as the change in bacterial localization over a 15 min timeframe [PAO1·μm^−2^·min^−1^]. For comparison across domains, the localization speed was then normalized by dividing by the sum of all localization speeds. Experiments with beads were analyzed like bacterial data.

## 3. Results

### 3.1. Binding and Subsequent Effects of the P. aeruginosa Strain PAO1 on Phase-Separated Lipid Bilayers Containing the Glycosphingolipid Gb3

Using supported lipid bilayers, we aimed to elucidate the initial interactions of the bacterium *P. aeruginosa* with the host cell glycosphingolipid Gb3, which have been reported to mainly trigger the internalization of the bacterium into host cells [6].

**Figure 1:**
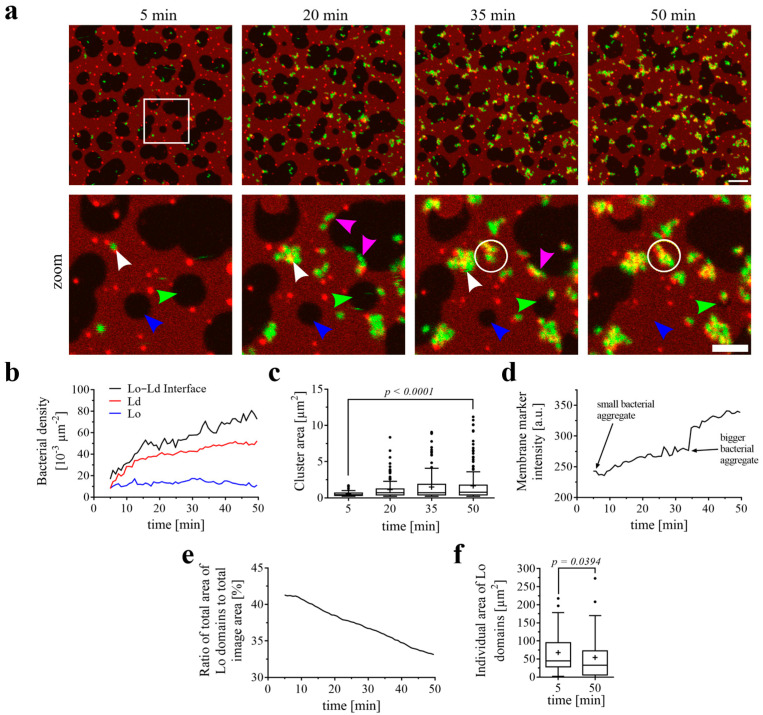
*P. aeruginosa* binding and effects on phase-separated SLBs containing a natural mixture of Gb3 species. The SLB composition was DOPC/Chol/SM/Gb3 (37.5/20/37.5/5 mol-%) with the supplement of 0.25 mol-% of the fluorescent lipid Texas Red-DHPE. (**a**) Representative time series of the interactions of the *P. aeruginosa* strain PAO1 (GFP-tagged; green) with a phase-separated SLB. Texas Red-DHPE (red) marks the Ld domains. The indicated time points represent the time after the addition of bacteria. The lower panel displays the zoomed-in area, which is highlighted by a white open square in the upper panel. Scale bars are 10 and 5 µm, respectively. The bacteria bound preferentially to the Lo–Ld interfaces (pink arrowheads point to representative examples). The bacteria aggregated at the SLB surface and the aggregates grew over time (white arrowhead points to a representative example). The bacterial aggregates accumulated lipids from the SLBs (white open circle highlights one representative example). Lo domains decreased in area size and completely disappeared (green arrowhead), even without direct contact with bacteria (blue arrowhead). (**b**) Change in density of *P. aeruginosa* on Ld domains, Lo domains, and at the Lo–Ld interfaces over time. (**c**) Change in bacterial cluster area over time. Means are indicated as “+”. (**d**) Change in membrane marker intensity. The averaged Texas Red signal collected from the area indicated by the white open circle in (**a**) is depicted over time. (**e**) The proportion of Lo domains (as a percentage of the total image area) decreased over time. (**f**) Area changes of individual Lo domains over time. Means are indicated as “+”. The full-time sequence is available as Supplementary Movie S1.

Plasma membranes exhibit asymmetrical lipid composition in the inner and outer leaflets. Phosphatidylcholine, sphingomyelin (SM), and glycosphingolipids are mainly found in the outer leaflet, while phosphatidylserine, phosphatidylethanolamine, and phosphatidylinositol are present in the inner leaflet, with cholesterol (Chol) being found in both leaflets [33,34,35,36]. In order to mimic the plasma membrane heterogeneity of the outer leaflet, we prepared SLBs on the mica surface using a lipid composition of DOPC, Chol, SM, and the glycosphingolipid Gb3 (37.5/20/37.5/5 mol-%). It is important to mention that Gb3 stands for a natural mixture of the glycosphingolipid Gb3 differing in its fatty acyl chain by length, hydroxylation, and degree of saturation. The lipid mixture was supplemented by the fluorescent lipid Texas Red-DHPE (0.25 mol-%, red color) that preferentially accumulated in Ld domains (in our SLBs on average approximately 86%), whereas Lo domains appeared rather dark (Figure 1a). We applied the chromosomally GFP-tagged *P. aeruginosa* PAO1 wild-type strain (15 × 10^6^ cells mL^−1^) to SLBs at room temperature and observed the interactions of fluorescent bacteria (green) with the membrane (red, Figure 1a and Supplementary Movie S1) for approximately 45 min. In the beginning, individual bacteria approached the SLB, but over time, more and more bacteria populated the membrane. Interestingly, bacteria were not uniformly distributed. They localized predominantly at the boundaries of the Ld and Lo domains (pink arrowheads point to representative examples). The bacterial density was always the highest at Lo–Ld interfaces, throughout the entire observation time (Figure 1a and corresponding quantifications in Figure 1b). For quantification, the interface between Lo and Ld domains was defined as 1 µm, similar to the size of a bacterial cell. Bacteria were then counted in each section and normalized to the entire image area. After approximately 10 min of incubation, bacteria began to form aggregates on the membrane surface with newly attaching bacteria contributing to the growth of these aggregates over time (Figure 1a,c, white arrowhead points to an example). Furthermore, the fluorescence intensity of the membrane marker Texas Red-DHPE increased over time at the sites where bacteria and bacterial aggregates attached, indicating a local enrichment of lipids. One representative example describing the events following the attachment of bacteria was selected (Figure 1a, white open circle) and quantitatively analyzed (Figure 1d). At this point, we speculate that *P. aeruginosa* tries to cover a maximum of its surface with membrane lipids, probably to destabilize the host cell membrane and trigger its cellular uptake, for instance, by the LecA-driven lipid zipper mechanism [6].

Simultaneously with the formation of bacterial aggregates, a drastic restructuring of the SLB occurred (Figure 1a). The entire proportion of Lo domains (Figure 1e) decreased from 41.3% of the entire image area to 33.1%, while the areas of individual Lo domains (Figure 1f) shrunk over time from an average of 67.8 µm^2^ to 54.4 µm^2^. Remarkably, several Lo domains dissolved and thus disappeared completely (Figure 1a, blue and green arrowheads point to representative examples). Interestingly, Lo domains that had never been in contact with a bacterium also dissolved over time (Figure 1a, blue arrowhead).

As previously published, Gb3 is a crucial receptor for the bacterium inducing membrane curvature, which leads to membrane invaginations and internalization of the bacterium into the host cells [6]. Since LecA plays a key role in the pathogenicity of *P. aeruginosa* and can reorganize and disrupt membranes [21], in a dose-dependent manner (Supplementary Figure S1 and Supplementary Movies S2–S6), we hypothesized that it might also play an important role in the initial bacterial binding and localization to the membrane. When Gb3-containing SLBs were incubated with bacteria in the presence of the glycomimetic *para*-nitrophenyl-α-D-galactopyranoside (PNPG, 10 mM), localization preferences were less pronounced, and localization to Ld was even higher than to Lo–Ld interfaces. PNPG saturates the carbohydrate-binding sites of LecA on the outer bacterial membrane and thereby interferes with the binding of the bacterium via LecA to the terminal α-galactose of the host cell glycosphingolipid Gb3 (Supplementary Figure S2 and Supplementary Movie S7). This also indicates that the interactions between LecA and Gb3 play a role in this phenomenon.

**Figure 2:**
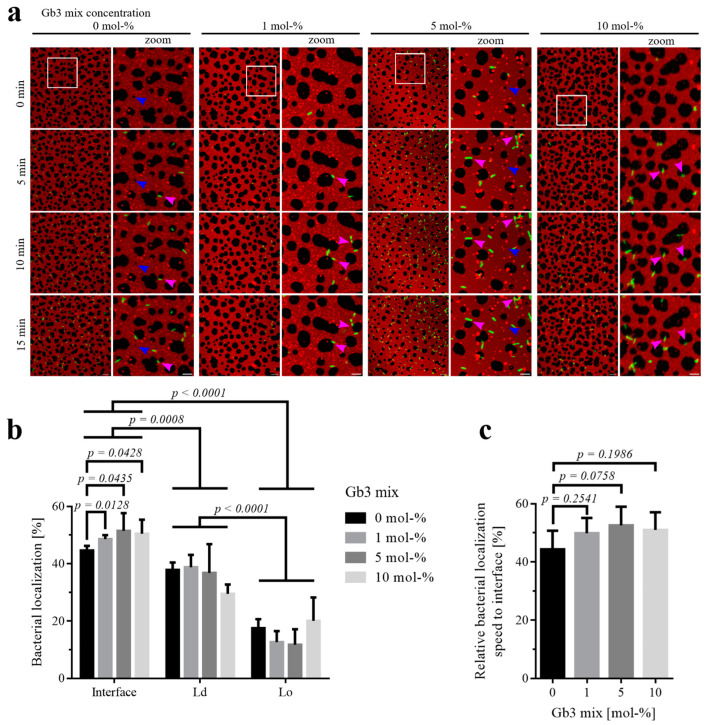
Early *P. aeruginosa* localization and relative localization speed on phase-separated SLBs containing different amounts of a natural mixture of Gb3 species. The SLBs were composed of the respective indicated Gb3 mix amount (0, 1, 5, 10 mol-%), 20 mol-% cholesterol, and equal parts of DOPC and SM (40, 39.5, 37.5, 35 mol-%), spiked with 0.25 mol-% of fluorescent Texas Red-DHPE. We analyzed the first 15 min of movies, as soon as five or more bacteria were detected in the frame. (**a**) Representative time series of the localization of the *P. aeruginosa* strain PAO1 (GFP-tagged; green) on SLBs (Texas Red-DHPE (red) marks the Ld domains). Next to the full-frame panels are zoomed-in areas, which are highlighted by white open squares in their respective full-frame images. Scale bars are 10 and 5 µm, respectively. Bacteria bound preferentially at the Lo–Ld interfaces (pink arrowheads point to representative examples). Lo domains decreased in area size and completely disappeared, even without direct contact with bacteria (blue arrowhead). (**b**) The quantification of bacterial localization (in %) on the Lo–Ld interface, Ld, and Lo domains as a function of Gb3 concentration on the SLBs. With an increasing concentration of Gb3 mix, bacterial localization towards the Lo–Ld interface increased. Independently of the concentration of Gb3 mix in the SLB, *P. aeruginosa* predominantly localized towards the domain interfaces, less so to the Ld domains, and least to the Lo domains (*n* ≥ 3). (**c**) Comparing the influence of the Gb3 mix amount in the SLBs on the relative localization speed of *P. aeruginosa* to Lo–Ld interfaces, compared to the individual domains, showed no significant difference between the samples, but the localization speed increased with higher Gb3 concentrations (*n* ≥ 3). The full-time sequences are available as Supplementary Movies S8–S11.

In order to investigate the early phase of interaction between bacteria and SLB, we analyzed 15 min time windows starting when at least five bacteria were detected in the vicinity of the membrane. This was conducted to ensure that the samples could be compared with each other, as the bacteria may arrive at the membrane at different time points during acquisition. Because the data are normalized to the image area, a statistically equal distribution towards Lo–Ld interfaces, Lo, and Ld domains would result in detecting one-third or 33.3% of bacteria in each area. We tested several concentrations of the Gb3 species mixture in SLBs (1, 5, or 10 mol-%), and all samples exhibited a significant bacterial localization towards the interface (48.6%, 51.5%, 50.5%, respectively) compared to the negative control without Gb3, which had an average value of 44.6% (Figure 2a,b, pink arrowheads, and Supplementary Movies S8–S11). Independently of the amount of Gb3 in the SLB, *P. aeruginosa* is located mainly towards the interface, less to Ld domains, and least to Lo domains (Figure 2b). We wondered if bacteria can locate and potentially bind faster to Lo–Ld interfaces of SLBs with higher Gb3 species mixture content. Hence, we analyzed the movies regarding the relative localization speed of bacteria towards interfaces compared to Ld or Lo domains. We therefore determined the change in bacterial localization at the interface over a 15 min timeframe. To compensate for variations in bacteria levels between different samples, we normalized the data by dividing the localization speeds by the sum of all localization speeds across the domains. The relative speed of the bacterial localization to interfaces increased with a higher receptor amount, from 44.3%, to 49.9%, to 52.6%, to 51.0% for 0, 1, 5, and 10 mol-%, respectively, but was not significant (Figure 2c). Additionally, it is important to note that when *P. aeruginosa* were added to SLBs devoid of the glycosphingolipid Gb3, bacteria also came down onto the SLB, exploring the membrane. Interestingly, *P. aeruginosa* were found majorly at the Lo–Ld interface with an average of 44.6% (Figure 2a, pink arrowheads, and Supplementary Movie S8), and domain dissolution could still be observed (Figure 2a, blue arrowheads).

### 3.2. Binding and Impact of the P. aeruginosa Strain PAO1 on Phase-Separated Lipid Bilayers Containing the Synthetic Lipid FSL-Gb3

The interaction between LecA and Gb3 appeared to play a role in the observed localization of *P. aeruginosa* to the Lo–Ld interfaces, albeit not being the sole cause. Several studies, which investigated the interactions of Gb3-binding lectins, e.g., the B-subunit of Shiga toxin (StxB) or the *P. aeruginosa* lectin LecA, with Gb3 (present as a natural mixture of Gb3 species) using synthetic membrane systems, have shown that these lectins mainly bound to Gb3 partitioning in Lo domains [37,38,39]. On the contrary, in this study, the *P. aeruginosa* PAO1 strain was observed to bind marginally to Lo domains, largely preferring the interface between Lo and Ld domains and, to a lesser extent, Ld domains (Figure 1b).

In order to investigate this phenomenon in more detail and from a different angle, we established SLBs supplemented with FSL-Gb3, a phospholipid-based synthetic Gb3 analog (Function-Spacer-Lipid with globotriose trisaccharide), which is almost exclusively incorporated into Ld domains [39]. LecA was able to reorganize SLBs containing FSL-Gb3 and dissolve Lo domains as well (Supplementary Figure S1e and Supplementary Movie S6). Can the effects described above for the Gb3 mixture be confirmed by using a single Gb3 species, or would bacteria only colonize in the Ld phase?

**Figure 3:**
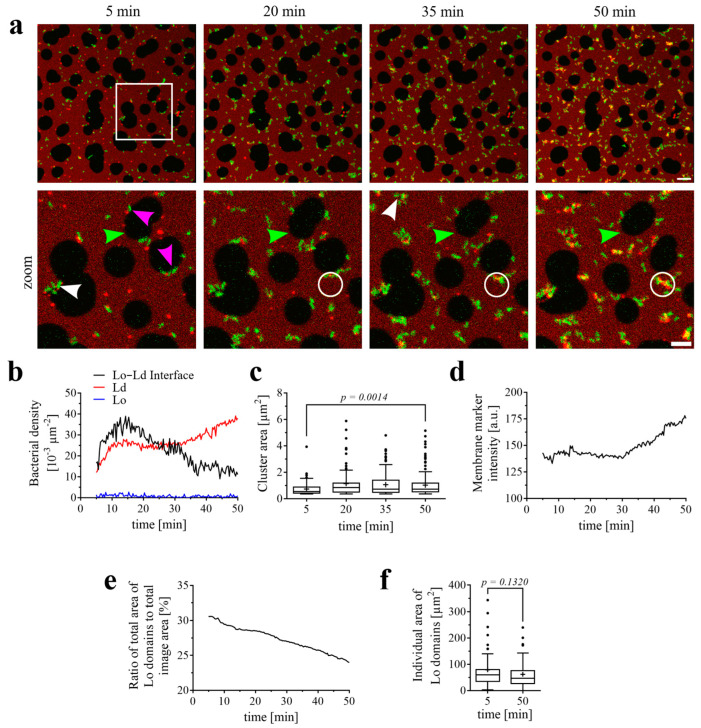
*P. aeruginosa* binding and effects on phase-separated SLBs containing the synthetic Gb3 analog FSL-Gb3. The SLB composition was DOPC/Chol/SM/FSL-Gb3 (37.5/20/37.5/5 mol-%) with the supplement of 0.25 mol-% of the fluorescent lipid Texas Red-DHPE. (**a**) Representative time series of the interactions of the *P. aeruginosa* strain PAO1 (GFP-tagged; green) with an SLB (Texas Red-DHPE (red) marks the Ld domains). The indicated time points represent the time after the addition of bacteria. The lower panel displays the zoomed-in area, which is highlighted by a white open square in the upper panel. Scale bars are 10 and 5 µm, respectively. Bacteria bound preferentially at the Lo–Ld interfaces at the early time points (pink arrowheads point to representative examples). The bacteria aggregated at the SLB surface and the aggregates grew over time (white arrowhead points to a representative example). The bacterial aggregates accumulated lipids from the SLBs (white circle highlights one representative example), and Lo domains decreased in size (green arrowhead). (**b**) Change in density of *P. aeruginosa* on Ld domains, Lo domains, and at the Lo–Ld interfaces over time. (**c**) Change in bacterial cluster area over time. Means are indicated as “+”. (**d**) Change in membrane marker intensity. The averaged Texas Red signal collected from the area indicated by the white open circle in (**a**) is depicted over time. (**e**) The total area of Lo domains (as a percentage of the total image area) decreased over time. (**f**) Area changes of individual Lo domains over time. Means are indicated as “+”. The full-time sequence is available as Supplementary Movie S12.

To answer this question, the *P. aeruginosa* strain PAO1 was applied to FSL-Gb3 containing SLBs at the same concentrations as for the experiments with SLBs containing a mixture of Gb3 species, as presented in Figure 1. Interestingly, a similar bacterial activity was observed at the surface of SLBs. The bacteria bound to the membrane via FSL-Gb3 and aggregated over time (Figure 3a and Supplementary Movie S12). Initially, the highest bacterial densities were measured at the Lo–Ld interfaces (pink arrowheads point to examples) like with the natural mixture of Gb3 species (Figure 1b). But after approximately 15 to 20 min, the bacterial density at Lo–Ld domain interfaces decreased in favor of increasing densities at Ld domains, while binding to Lo domains became negligible (Figure 3b). We observed the early formation of bacterial aggregates, which grew over time (white arrowheads point to examples) and saturated at later time points (Figure 3a,c). Here, too, a local enrichment of membrane lipids was recorded, indicated by an increase in the fluorescence intensity of the membrane marker Texas Red-DHPE over time (Figure 3d, statistics originate from the white circle in Figure 3a). Lo domains also decreased in size (green arrowheads point to example). The total area of Lo domains (Figure 3e) was reduced over time from 30.6% of the image area to 24.0%. The areas of individual Lo domains (Figure 3f) shrunk as well, from an average of 78.8 µm^2^ to 61.9 µm^2^.

*P. aeruginosa* did not accumulate on the Lo membrane domains, devoid of Gb3, supporting the role of Gb3 in localization and binding. The bacteria succeeded in sensing and initially targeting the boundary region between co-existing lipid phases as indicated by the highest bacterial densities recorded at Lo–Ld interfaces. Similar localization phenomena at domain interfaces have been reported for several enveloped viruses [40,41,42,43], which are much smaller particles.

### 3.3. Binding and Effects of LecA-Coated Beads on Phase-Separated Lipid Bilayers Containing Either the Natural Mixture of Gb3 Species or the Synthetic Lipid FSL-Gb3

The main interaction partner of the host cell glycosphingolipid Gb3 on the part of the bacterium is the α-galactose-specific lectin LecA. We have previously demonstrated the ability of LecA to bind to various Gb3 species in different membrane environments and to trigger membrane reorganization, leading in part to the dispersion of Lo domains [21,39]. Furthermore, it was shown that LecA-induced Gb3 clustering, referred to as “lipid zipper”, results in negative membrane curvature of the plasma membrane and ultimately leads to the cellular uptake of the bacterium [6,17].

Therefore, it is of great interest to analyze the impact of the *P. aeruginosa* lectin LecA at lipid phase boundaries. To keep the approximate dimensions of the bacterium and to study the exclusive effects of LecA, we chose LecA-coated silica beads (with a diameter of 1 µm), which have already proven successful in other studies with cells and giant unilamellar vesicles [31,32].

**Figure 4:**
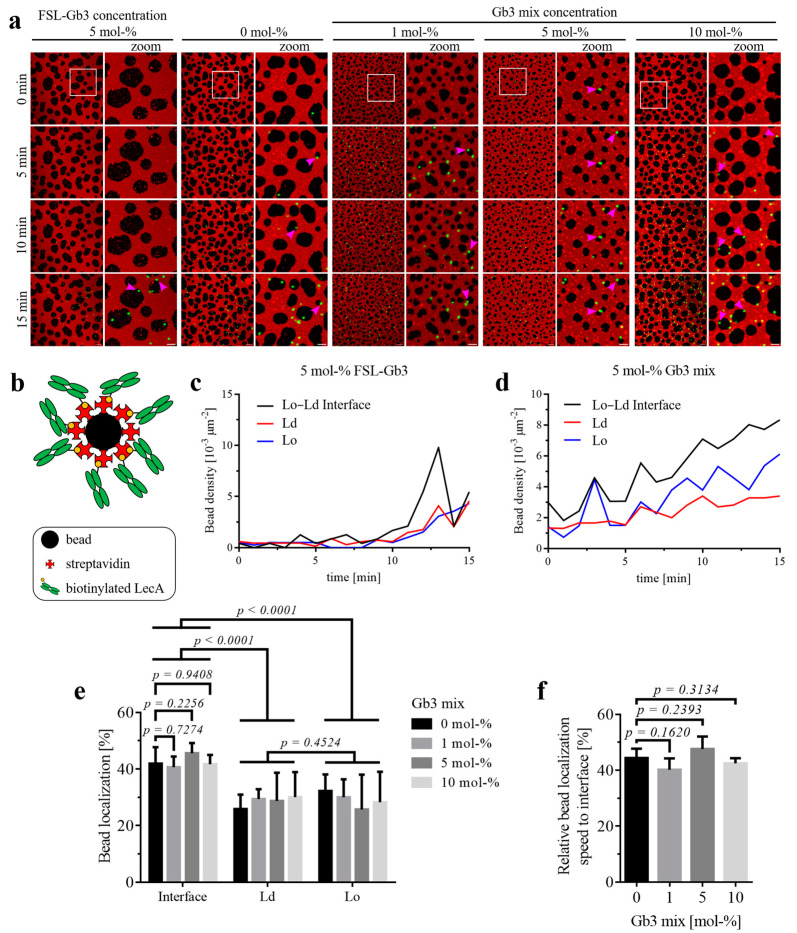
Binding and interactions of LecA-coated beads with phase-separated SLBs. The SLBs consisted of the indicated synthetic Gb3 analog FSL-Gb3 (5 mol-%) or the natural mixture of Gb3 species at various concentrations (0, 1, 5, 10 mol-%), 20 mol-% cholesterol, and equal parts of DOPC and SM (40, 39.5, 37.5, 35 mol-%), doped with 0.25 mol-% of fluorescent Texas Red-DHPE. We analyzed the first 15 min of movies after five or more beads were detectable in the frame. (**a**) Representative time series of the localization of the LecA-coated beads (far-red fluorescent; depicted in green) on SLBs. The lipid Texas Red-DHPE (red) was used as a membrane marker, which partitions in the Ld phase. Next to the full-frame panels are zoomed-in areas, which are highlighted by white open squares in their respective full-frame images. Pink arrowheads point to examples of bacteria binding to Lo–Ld domain interfaces. Scale bars are 10 and 5 µm, respectively. (**b**) Illustration of a streptavidin microsphere with a diameter of 1 µm, and LecA coupled to it via biotin (for illustration purposes, not to scale). (**c**,**d**) For this representative time series, the changes in bead density are illustrated over time on Ld domains, Lo domains, and at Lo–Ld interfaces of SLBs containing the synthetic Gb3 analog FSL-Gb3 (**c**) and the natural mixture of Gb3 species (**d**). (**e**) The difference in localization of LecA-coated beads towards the Lo–Ld interface over an increasing concentration of the natural mixture of Gb3 species was not significant. Independently of the concentration of the Gb3 mixture in the SLB, LecA-coated beads mainly located towards the domain interfaces, less to Ld and Lo domains (*n* ≥ 4). (**f**) A comparison of the influence of the concentration of the Gb3 mixture in the SLBs on the relative localization speed of LecA-coated beads at Lo–Ld interfaces, compared to the individual domains, revealed no significant difference between these samples (*n* ≥ 4). The full-time sequences are available as Supplementary Movies S13–S17.

Here, we investigated the early interaction phase of LecA-coated beads on SLBs with the synthetic Gb3 analog FSL-Gb3 and various concentrations of the natural mixture of Gb3 species (0, 1, 5, or 10 mol-%), similar to the experiments with bacteria (Figure 4a and Supplementary Movies S13–S17). For this purpose, time windows of 15 min were analyzed as soon as five or more LecA-coated beads were detected in the imaging area. In both cases, the LecA-coated beads (Figure 4b, as a not to scale illustration) bound to Gb3 but failed in inducing additional effects that have been described for bacteria, such as aggregation, lipid accumulation, as well as shape changes and dispersion of Lo domains. For SLBs containing FSL-Gb3, the bead density was relatively equal at the Lo–Ld interfaces and the Ld and Lo domains (Figure 4c), with some preference for the interface after 10 min. Interestingly, LecA-coated beads bound preferentially to the Lo–Ld phase boundaries of SLBs (pink arrowheads in Figure 4a point to examples) containing the natural mixture of Gb3 species (Figure 4d). For the beads, increasing the Gb3 concentration in the SLBs did not lead to a significant increase in localization at the Lo–Ld interface after 15 min. A higher concentration of Gb3 had no significant effect on the localization of the LecA-coated beads at the Lo–Ld interface (Figure 4e), while all samples showed that the localization at the interface was significantly higher compared to the Ld or Lo domains (Figure 4e). We investigated whether LecA brings the beads to the Lo–Ld interface faster than the domains themselves when applied to SLBs with a higher concentration of the natural mixture of Gb3 species. The analysis revealed that the relative localization speed of LecA-coupled beads at the interface was not significantly affected by the Gb3 concentration of the SLBs (Figure 4f).

Control beads that were not functionalized with LecA showed similar behavior, as they also localized at the Lo–Ld interface (Supplementary Figure S3a and Supplementary Movies S18 and S19). As with LecA-coupled beads, the localization of control beads to domain interfaces was significant, regardless of receptor concentration. Comparing the bead localization at Lo–Ld interfaces showed no significant differences between SLBs with or without 5 mol-% of Gb3 (Supplementary Figure S3b). The speed at which the control beads localized at the interface also did not differ significantly between the samples with or without 5 mol-% of Gb3 (Supplementary Figure S3c).

### 3.4. Localization and Subsequent Effects of the P. aeruginosa PAO1 LecA Knockout Mutant Strain on Phase-Separated Lipid Bilayers Containing the Glycosphingolipid Gb3

**Figure 5:**
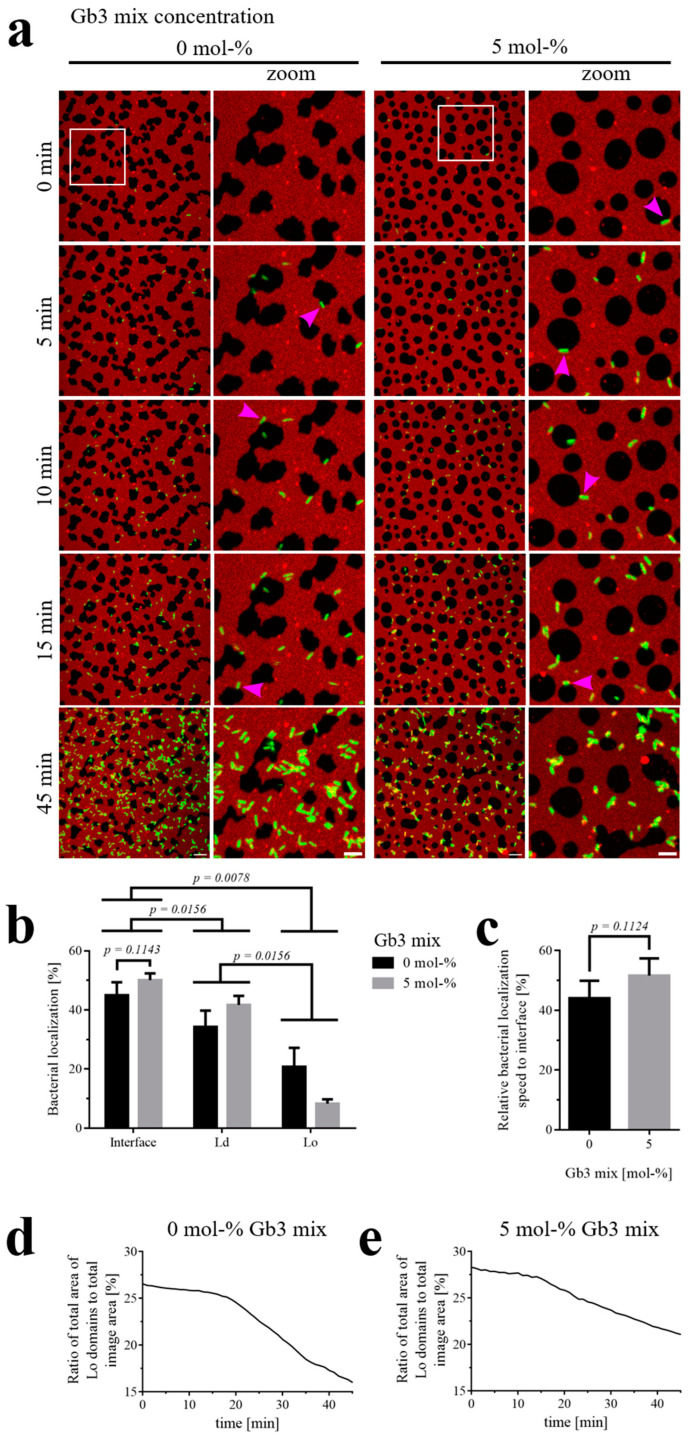
Localization of the *P. aeruginosa* LecA knockout mutant and effects on phase-separated SLBs containing a natural mixture of Gb3 species. The SLBs were composed of the respective indicated Gb3 mix amount (0, 5 mol-%), 20 mol-% cholesterol, and equal parts of DOPC and SM (40, 37.5 mol-%), spiked with 0.25 mol-% of fluorescent Texas Red-DHPE. (**a**) Representative time series of the localization of the *P. aeruginosa* strain PAO1 with LecA knocked out (GFP-tagged; green) on SLBs (Texas Red-DHPE (red) marks the Ld domains). Next to the full-frame panels are zoomed-in areas, which are highlighted by white open squares in their respective full-frame images. Scale bars are 10 and 5 µm, respectively. Bacteria bound preferentially at the Lo–Ld interfaces (pink arrowheads point to representative examples). (**b**) The quantification of bacterial localization (in %) to the Lo–Ld interface, Ld, and Lo domains as a function of Gb3 concentration on the SLBs after 15 min. With a 5 mol-% Gb3 mix, bacterial localization towards the Lo–Ld interface increased, although not significantly. Independently of the Gb3 mix in the SLB, *P. aeruginosa* predominantly localized towards the domain interfaces, less so to the Ld domains, and least to the Lo domains (*n* = 4). (**c**) Comparing the influence of the Gb3 mix amount in the SLBs on the early relative localization speed of *P. aeruginosa* to Lo–Ld interfaces, compared to the individual domains, showed no significant difference between the samples, although the localization speed increased with higher Gb3 concentrations (*n* = 4). (**d**,**e**) The proportion of Lo domains (as a percentage of the total image area) decreased over time for SLBs without or with 5 mol-% Gb3 mix. The full-time sequences are available as Supplementary Movies S20 and S21.

Since the impact of LecA and its interaction with Gb3 during its localization on SLBs remained unclear, we utilized a LecA knockout mutant of the *P. aeruginosa* strain PAO1 (GFP-tagged) to further elucidate the role of LecA during the early interaction phase. For that, we analyzed the interactions of the LecA knockout bacterium on SLBs containing either 0 or 5 mol-% of the natural mixture of Gb3 species (Figure 5a and Supplementary Movies S20 and S21). Looking at the early time window, when bacterial cells initially arrived at the membrane, they were located mainly towards the Lo–Ld domain interfaces (pink arrowheads in Figure 5a point to examples), less to Ld domains, and least towards Lo domains, independently of the presence of Gb3 mix in the SLB (Figure 5b). Comparing the localization to the Lo–Ld domain interfaces in regard to the Gb3 mix showed an increase in localization towards these interfaces (average of 45.0 % for 0 mol-% Gb3 mix, 50.1 % for 5 mol-% Gb3 mix), albeit not being significant. Additionally, we checked, if the presence of 5 mol-% Gb3 mix let *P. aeruginosa* locate to Lo–Ld domain interfaces faster compared to the individual domains. We found that the relative localization speed of the bacteria was not significantly increased with the Gb3 mix (Figure 5c). The wild-type *P. aeruginosa* strain was able to reorganize the membrane and dissolve Lo domains even in the absence of the Gb3 mix in the membrane (Figure 2a). Therefore, we also examined changes in the size of Lo domains when the LecA knockout mutant was applied to SLBs. The Lo domain area decreased distinctly from 26.5 % of the image area to 16.0 % over the course of 45 min without the Gb3 mix present (Figure 5d). At the same time, for the SLB containing 5 mol-% Gb3 mix, the Lo domain area shrunk from 28.3 % to 21.1 % (Figure 5e), all in all showing the ability of *P. aeruginosa* to reorganize the membrane without LecA as well.

Overall, despite the initial assumption that LecA might be the key factor directing *P. aeruginosa* PAO1 to domain interphases, it was eventually found that the locally occurring Gb3 appears to play a greater role than LecA. Nevertheless, we could detect a minor influence of LecA when looking at the wild-type and comparing it to the LecA knockout mutant. However, it seems that other bacterial factors, possibly different lectins, unspecific interactions with Gb3, or possibly physical effects may also play a role. On top of that, even though free LecA is capable of reorganizing membranes drastically, when Gb3 is present (Supplementary Figure S1), the LecA knockout mutant of *P. aeruginosa* showed its great ability to do this in a similar way without LecA.

## 4. Discussion

We utilized SLBs to investigate the interactions of the highly critical pathogen *P. aeruginosa* (strain PAO1) with Gb3-containing phase-separated membranes, mimicking lipid raft-like structures of host cells. Using this synthetic membrane system, we were able to show that *P. aeruginosa* binds preferentially to the interfaces of Lo and Ld domains, then to a lesser extent to Ld domains, and least to Lo domains, although it is known that one of its receptors, the glycosphingolipid Gb3, was mainly found in Lo domains [37,38,39]. Since micro- and nanodomains of human cells contain many important proteins and receptors like CD59 and Gb3, which form signaling platforms, they are directly targeted by pathogens like *P. aeruginosa* [20,44,45]. Interestingly, these Lo domains appear to be important not only because of their composition but also because of the nature of the plasma membrane itself. In particular, there is a height deviation of about 0.8 nm between the SM/Chol-rich Lo domains and the mainly DOPC-containing Ld domains, which is partly due to differences in the acyl chain length of the lipids [46]. This state is energetically unstable, so that the boundary between different membrane domains represents a weak point in the membranes that is targeted by viral and bacterial pathogens [40,42,43,47,48]. For example, the fusion of HIV is facilitated by its gp41 protein at membrane phase boundaries [42,43]. It is known from the bacterial world that the pore-forming toxin α-hemolysin of *E. coli* also initially binds to membrane domain interfaces and finally accumulates in the Lo domain [47]. We were able to show that *P. aeruginosa* targets these vulnerable sites as well and, thus, further promotes its virulence, presumably finding a suitable place to enter the host cell. Targeting the domain interfaces could be partly attributed to a physical effect. Similar observations were previously made with quantum dots and simulations of gold nanoparticles, which were also preferentially located at phase boundaries [49,50]. Simulations revealed that the adsorption of these cationic nanoparticles was promoted by their interactions with the membrane lipids, as well as the innate membrane surface curvature between Lo and Ld domains. The increased rigidity of Lo domains makes it more difficult to bend the membrane, which favors localization at the Ld domain and interfaces. Although the quantum dots were only about 7–9 nm in diameter and the simulations were performed with 4 nm gold nanoparticles, both of which are much smaller than a 1 µm bacterium, comparable explanations could be given for the reported localization preference of the control beads without LecA or *P. aeruginosa*. Since LecA induces a slight inward bending of the membrane, the binding of LecA as well as the entire bacterium to domain boundaries could be energetically favored in a similar way [6,17]. Another influence on the preference of *P. aeruginosa* to bind to interfaces could be the accessibility of LecA receptor Gb3 [51]. The dense packing of the Lo domains could have an influence on the orientation of the receptor, especially the trisaccharide headgroup, and thus affect LecA interactions and general bacterial adhesion [52]. Surprisingly, the local Gb3 concentration seems to influence bacterial localization more than the presence of LecA, since the localization of wild-type and LecA-knockout mutant both increased with higher Gb3 concentration. We speculate that Gb3 might also interact with bacteria in an unspecific way, whereby Gb3 is more accessible on the border of Lo domains, compared to the inner areas of Lo domains, which makes these interactions easier.

Intriguingly, these domain boundaries are not only important in the context of pathogens but also for the transport of enzymes. For instance, the translocation of the Rho-GTPase Rac1 to the membrane happened at domain interfaces in an SLB system with subsequent diffusion and accumulation in the Ld phase [53].

We also report the reorganization of the Lo and Ld domains with concomitant lipid accumulation at the bacterial adhesion site. We hypothesize that *P. aeruginosa*, whose LecA is bound to the outside, wraps itself with membrane lipids, possibly attempting to promote the invasion process [16]. Lipid accumulation has also been observed for LecA-driven adhesion of the *P. aeruginosa* PAO1 strain to Gb3-containing spherical giant unilamellar vesicles, suggesting that it is not an artifact caused by the use of a planar-supported membrane system that prevents natural membrane deformations [6]. Furthermore, bacterial domain reorganization could bear another advantage for the pathogen. By dissolving the rather rigid Lo domains, entry through the membrane could be easier for the bacterium. Although free LecA is able to reorganize membranes and dissolve Lo domains, it is not the only responsible bacterial agent that triggers these phenomena, as we could observe these effects for the LecA-knockout mutant strain as well. Follow-up studies should clarify whether LecA-induced effects only occur at a certain density of bacteria or LecA-coated beads, or whether a certain amount of soluble LecA near the membrane surface is required. In contrast to beads where LecA is linked via strong streptavidin–biotin interactions, LecA is bound to the bacterium via weak carbohydrate–lectin interactions, which could favor the release of LecA from the bacterial outer membrane at any time point. In addition, Song et al. identified the quorum-sensing metabolite *N*-(3-oxo-dodecanoyl) homoserine lactone from *P. aeruginosa* as one of its responsible players in the dissolution of lipid domains in eukaryotic membranes [10]. Therefore, knockout mutants of *P. aeruginosa*, where other known membrane reorganizing factors like *N*-(3-oxo-dodecanoyl) homoserine lactone are deleted, could shine a light on the actual impact of LecA in this context.

## 5. Conclusions

Overall, we were able to show that *P. aeruginosa* is located specifically at the interfaces between the Lo and Ld domains of SLBs during its adhesion to membranes. This localization is more impacted by the local Gb3 concentration in the SLB than by its lectin LecA. Beads were also located at the boundaries of Lo–Ld domains. In contrast to LecA-coated beads, *P. aeruginosa* additionally exhibited bacterial aggregation on the bilayer, together with lipid accumulation and a general reorganization of the membrane, followed by dissolution of the Lo domains. The targeting of domain interfaces, as already reported for some viruses (e.g., HIV) or bacteria (e.g., *E. coli*) as well as for non-pathogenic proteins such as the Rho-GTPase Rac1, could be a general strategy of molecules to gain access to membranes [42,43,47,53].

## Data Availability

The data supporting this study are available from the corresponding authors upon reasonable request.

## Supplementary Materials

The following supporting information can be downloaded at https://www.mdpi.com/article/10.3390/biom15030341/s1, Figure S1: LecA binding and effects on SLBs containing the glycosphingolipid Gb3 mix or FSL-Gb3; Figure S2: *P. aeruginosa* binding and effects on SLBs containing Gb3 mix in the presence of 10 mM PNPG; Figure S3: Microsphere localization and effects on SLBs containing the glycosphingolipid Gb3 mix; Movie S1: *P. aeruginosa* binding and effects on a phase-separated SLB containing a natural mixture of Gb3 species; Movie S2: LecA binding and effects on a phase-separated SLB containing 0 mol-% Gb3 mix; Movie S3: LecA binding and effects on a phase-separated SLB containing 1 mol-% Gb3 mix; Movie S4: LecA binding and effects on a phase-separated SLB containing 5 mol-% Gb3 mix; Movie S5: LecA binding and effects on a phase-separated SLB containing 10 mol-% Gb3 mix; Movie S6: LecA binding and effects on a phase-separated SLB containing 5 mol-% FSL-Gb3; Movie S7: *P. aeruginosa* binding and effects on a phase-separated SLB containing 5 mol-% Gb3 mix in presence of 10 mM PNPG; Movie S8: Early *P. aeruginosa* localization and effects on a phase-separated SLB containing 0 mol-% Gb3 mix; Movie S9: Early *P. aeruginosa* localization and effects on a phase-separated SLB containing 1 mol-% Gb3 mix; Movie S10: Early *P. aeruginosa* localization and effects on a phase-separated SLB containing 5 mol-% Gb3 mix; Movie S11: Early *P. aeruginosa* localization and effects on a phase-separated SLB containing 10 mol-% Gb3 mix; Movie S12: *P. aeruginosa* binding and effects on a phase-separated SLB containing the synthetic Gb3 analog FSL-Gb3; Movie S13: Binding and interactions of LecA-coated beads with a phase-separated SLB containing the synthetic Gb3 analog FSL-Gb3; Movie S14: Binding and interactions of LecA-coated beads with a phase-separated SLB containing 0 mol-% Gb3 mix; Movie S15: Binding and interactions of LecA-coated beads with a phase-separated SLB containing 1 mol-% Gb3 mix; Movie S16: Binding and interactions of LecA-coated beads with a phase-separated SLB containing 5 mol-% Gb3 mix; Movie S17: Binding and interactions of LecA-coated beads with a phase-separated SLB containing 10 mol-% Gb3 mix; Movie S18: Microsphere localization and effects on a phase-separated SLB containing 0 mol-% Gb3 mix; Movie S19: Microsphere localization and effects on a phase-separated SLB containing 5 mol-% Gb3 mix; Movie S20: *P. aeruginosa* LecA knockout mutant localization and effects on a phase-separated SLB containing 0 mol-% Gb3 mix; Movie S21: *P. aeruginosa* LecA knockout mutant localization and effects on a phase-separated SLB containing 5 mol-% Gb3 mix.

## Abbreviations

The following abbreviations are used in this manuscript:

Abl	Abelson tyrosine kinase
Chol	Cholesterol
DHPE	1,2-dihexadecanoyl-sn-glycero-3-phosphoethanolamine
DOPC	1,2-dioleoyl-sn-glycero-3-phosphocholine
FSL	Functional-spacer-lipid
Gb3	Globotriaosylceramide
Gb3 mix	Natural mixture of Gb3 species
GFP	Green fluorescent protein
GPI	Glycophosphatidylinositol
h	Hour
HABA	4′-hydroxyazobenzene-2-carboxylic acid
Ld	Liquid-disordered
Lo	Liquid-ordered
min	Minute(s)
PBS	Phosphate-buffered saline
PNPG	Para-nitrophenyl-α-D-galactopyranosid
SLB	Supported lipid bilayer
SM	Porcine brain-sphingomyelin
StxB	B-subunit of Shiga toxin
