# Supplementary material for: The Bacterium P. aeruginosa Disperses Ordered Membrane Domains by Targeting Phase Boundaries
Source: Biomolecules. 2025 Feb 27;15(3):341. doi: 10.3390/biom15030341 (PMC11940534; doi:10.3390/biom15030341)
Supplement: Supplementary file 1 [file biomolecules-15-00341-s001.zip › Supplementary Figures.pdf]

Supplementary Figures

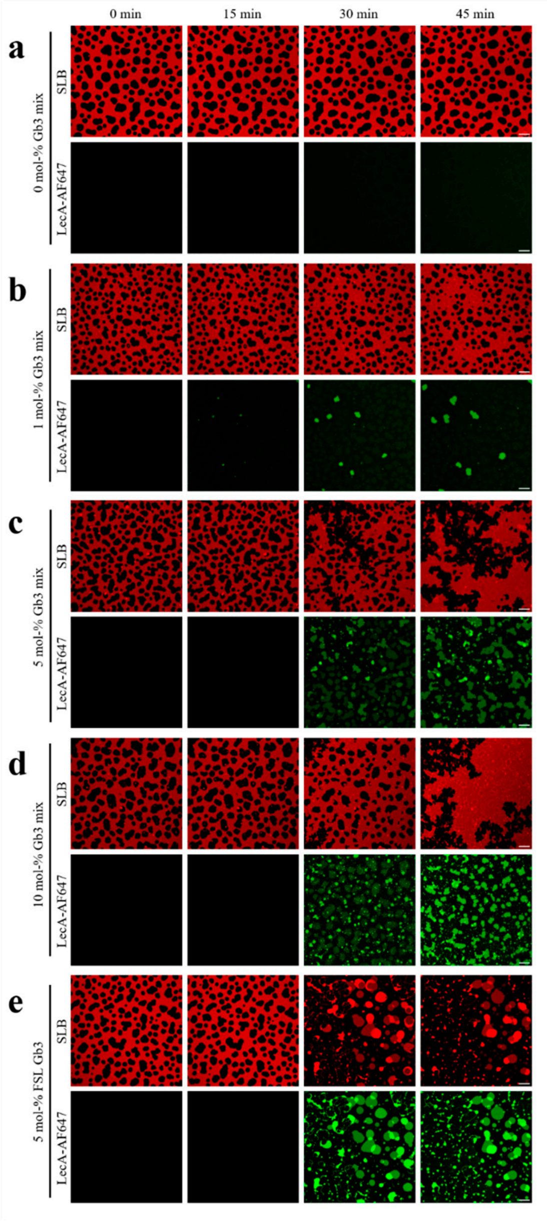

Figure S1. LecA binding and effects on SLBs containing the glycosphingolipid Gb3 mix or FSL-Gb3. The SLBs were comprised of the respective indicated Gb3 mix or FSL-Gb3 amount (0, 1, 5, 10 mol-%), 20 mol-% cholesterol, and equal parts of DOPC and SM (40, 39.5, 37.5, 35 mol-%), spiked with 0.25 mol-% of fluorescent Texas Red-DHPE. Representative time series of the interactions of 200 nM LecA (AlexaFluor647-tagged; depicted in green) with SLBs (Texas Red-DHPE (red) marks the Liquid-disordered (Ld) domains). The indicated time points represent the time after the addition of LecA. Scale bars are 10 µm. (**a**) LecA was unable to bind to SLBs devoid of Gb3. Lo domains were stable without LecA binding. (**b**–**d**) The presence of Gb3 mix enables binding of LecA to the membrane in a dose-dependent manner. 1 mol-% Gb3 mix exhibited membrane reorganization without its disruption, while the SLBs with higher Gb3 mix amounts were reorganized and dissolved after LecA binding. (**e)** The binding of LecA to FSL-Gb3 reorganized and disrupted SLBs even more compared to SLBs containing Gb3 mix. The full-time sequences are available as Supplementary Movies S2–S6.

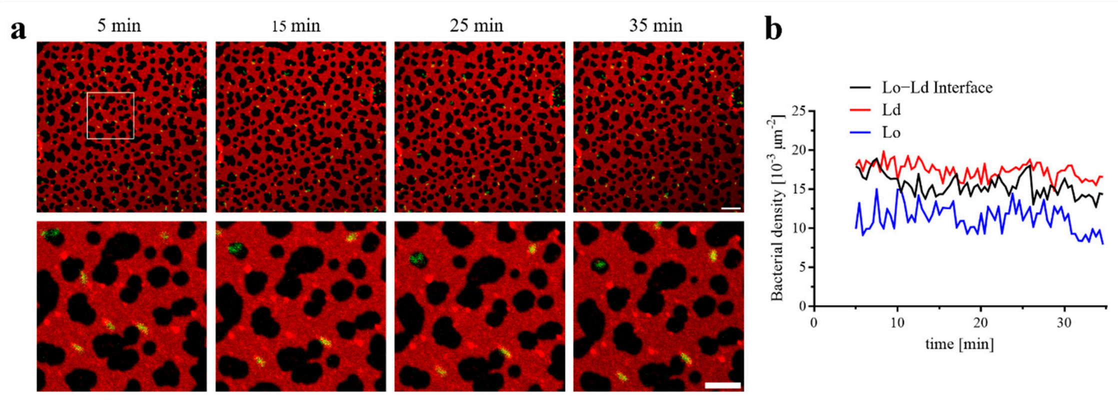

Figure S2. ***P. aeruginosa*** **binding and effects on SLBs containing Gb3 mix in the presence of 10 mM PNPG.** The SLB composition was DOPC/Chol/SM/Gb3 (37.5/20/37.5/5 mol-%) with the supplement of 0.25 mol-% of the fluorescent lipid Texas Red-DHPE. (**a**) Representative time series of the interactions of the *P. aeruginosa* strain PAO1 (GFP-tagged; green) with a SLB (Texas Red-DHPE (red) marks the Liquid-disordered (Ld) domains). The indicated time points represent the time after the addition of bacteria. The lower panel displays the zoomed-in area, which is highlighted by a white open square in the upper panel. Scale bars are 10 and 5 µm, respectively. The inhibition of LecA by 10 mM PNPG affected *P. aeruginosa* PAO1's localization. Additionally, domain sizes still decreased in size, albeit less drastically. (**b**) Change in density of *P. aeruginosa* on Ld domains, Lo domains and at the Lo-Ld interfaces over time. The full-time sequence is available as Supplementary Movie S7.

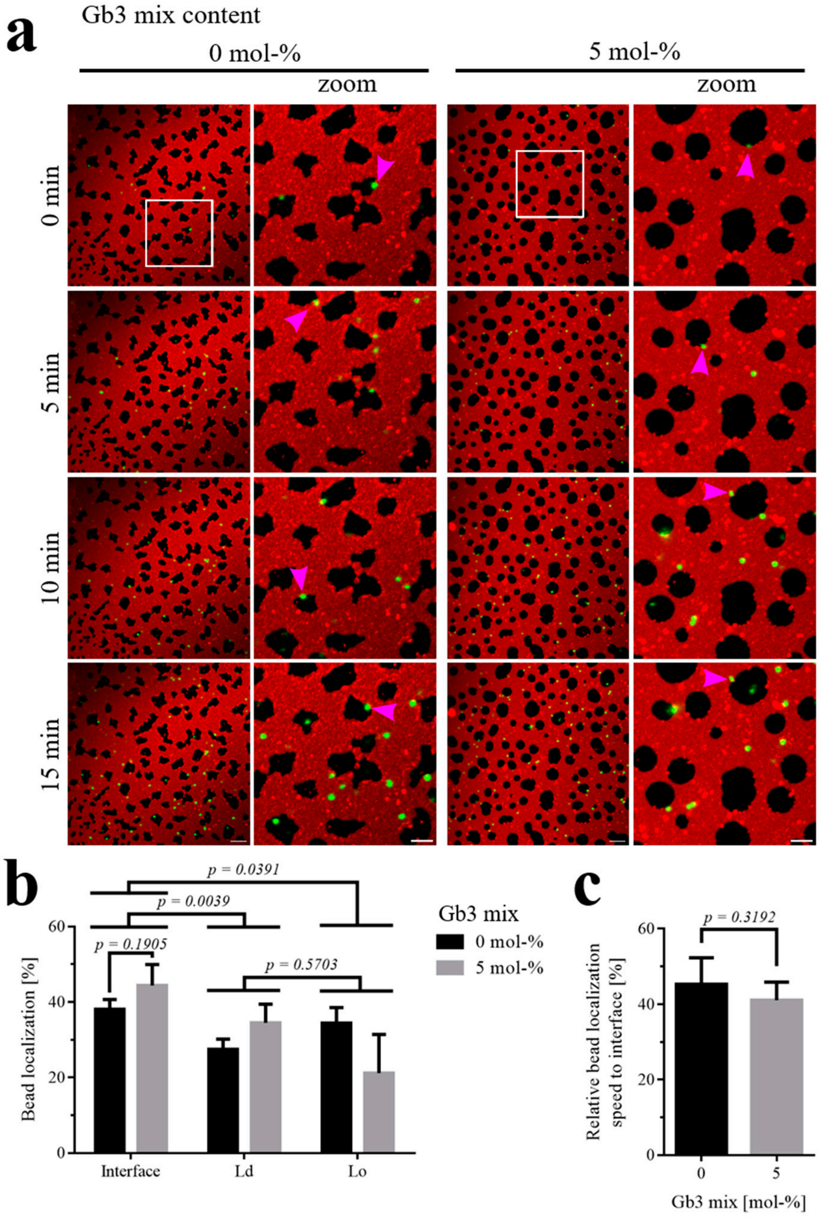

Figure S3. Microsphere localization and effects on SLBs containing the glycosphingolipid Gb3 mix. The SLBs were comprised of the respective indicated Gb3 mix amount (0, 5 mol-%), 20 mol-% cholesterol, and equal parts of DOPC and SM (40, 37.5 mol-%), spiked with 0.25 mol-% of fluorescent Texas Red-DHPE. We analyzed the first 15 min of movies, after 5 or more beads were detectable in the frame. (**a**) Representative time series of the localization of the beads (far-red fluorescent; depicted in green) on SLBs (Texas Red-DHPE (red) marks the Ld domains). Next to the full-frame panels are zoomed-in areas, which are highlighted by white open squares in their respective full-frame images. Scale bars are 10 and 5 µm, respectively. Beads preferentially located towards the Lo-Ld interfaces (pink arrowheads point to representative examples). (**b**) The difference of localization of beads towards the Lo-Ld interface over an increasing Gb3 mix amount was not significant. Regardless of presence of Gb3 mix, beads mainly located towards the domain interfaces, less to Ld domains and Lo domains ($n \geq 4$). (**c**) Comparing the influence of the Gb3 mix on the relative localization speed of beads to Lo-Ld interfaces, compared to the individual domains, showed no significant difference between those samples ($n \geq 4$). The full-time sequences are available as Supplementary Movies S18–S19.
