# Supplementary material for: The Bacterium P. aeruginosa Disperses Ordered Membrane Domains by Targeting Phase Boundaries
Source: Biomolecules. 2025 Feb 27;15(3):341. doi: 10.3390/biom15030341 (PMC11940534; doi:10.3390/biom15030341)
Supplement: Supplementary file 1 [file biomolecules-15-00341-s001.zip › Supplementary Movies/Supplementary Movie Descriptions.pdf]

Supplementary Movie Descriptions

Movie S1: *P. aeruginosa* binding and effects on a phase-separated SLB containing a natural mixture of Gb3 species. Representative movie of the interactions of the *P. aeruginosa* strain PAO1 (GFP-tagged; green) with a phase-separated SLB. Texas Red-DHPE (red) marks the Liquid-disordered (Ld) domains. The SLB composition was DOPC/Chol/SM/Gb3 (37.5/20/37.5/5 mol-%) with the supplement of 0.25 mol-% of the fluorescent lipid Texas Red-DHPE. The scale bar equals 10 µm.

Movie S2: LecA binding and effects on a phase-separated SLB containing 0 mol-% Gb3 mix. Representative movie of the interactions of LecA (AF488-tagged; green) with a phase-separated SLB. Texas Red-DHPE (red) marks the Ld domains. The SLB composition was DOPC/Chol/SM (40/20/40 mol-%) with the supplement of 0.25 mol-% of the fluorescent lipid Texas Red-DHPE. The scale bar equals 10 µm.

Movie S3: LecA binding and effects on a phase-separated SLB containing 1 mol-% Gb3 mix. Representative movie of the interactions of LecA (AF488-tagged; green) with a phase-separated SLB. Texas Red-DHPE (red) marks the Ld domains. The SLB composition was DOPC/Chol/SM/Gb3 (39.5/20/39.5/1 mol-%) with the supplement of 0.25 mol-% of the fluorescent lipid Texas Red-DHPE. The scale bar equals 10 µm.

Movie S4: LecA binding and effects on a phase-separated SLB containing 5 mol-% Gb3 mix. Representative movie of the interactions of LecA (AF488-tagged; green) with a phase-separated SLB. Texas Red-DHPE (red) marks the Ld domains. The SLB composition was DOPC/Chol/SM/Gb3 (37.5/20/37.5/5 mol-%) with the supplement of 0.25 mol-% of the fluorescent lipid Texas Red-DHPE. The scale bar equals 10 µm.

Movie S5: LecA binding and effects on a phase-separated SLB containing 10 mol-% Gb3 mix. Representative movie of the interactions of LecA (AF488-tagged; green) with a phase-separated SLB. Texas Red-DHPE (red) marks the Ld domains. The SLB composition was DOPC/Chol/SM/Gb3 (35/20/35/10 mol-%) with the supplement of 0.25 mol-% of the fluorescent lipid Texas Red-DHPE. The scale bar equals 10 µm.

Movie S6: LecA binding and effects on a phase-separated SLB containing 5 mol-% FSL-Gb3. Representative movie of the interactions of LecA (AF488-tagged; green) with a phase-separated SLB. Texas Red-DHPE (red) marks the Ld domains. The SLB composition was DOPC/Chol/SM/FSL-Gb3 (37.5/20/37.5/5 mol-%) with the supplement of 0.25 mol-% of the fluorescent lipid Texas Red-DHPE. The scale bar equals 10 µm.

Movie S7: *P. aeruginosa* binding and effects on a phase-separated SLB containing 5 mol-% Gb3 mix in presence of 10 mM PNPG. Representative movie of the interactions of the *P. aeruginosa* strain PAO1 (GFP-tagged; green) with a phase-separated SLB in presence of 10 mM PNPG. Texas Red-DHPE (red) marks the Ld domains. The SLB composition was DOPC/Chol/SM/Gb3 (37.5/20/37.5/5 mol-%) with the supplement of 0.25 mol-% of the fluorescent lipid Texas Red-DHPE. The scale bar equals 10 µm.

Movie S8: Early *P. aeruginosa* localization and effects on a phase-separated SLB containing 0 mol-% Gb3 mix. Representative movie of the early interactions of the *P. aeruginosa* strain PAO1 (GFP-tagged; green) with a phase-separated SLB. Texas Red-DHPE (red) marks the Ld domains. The SLB composition was DOPC/Chol/SM (40/20/40 mol-%) with the supplement of 0.25 mol-% of the fluorescent lipid Texas Red-DHPE. The movie shows the first 15 min after at least 5 bacteria were detectable. The scale bar equals 10 µm.

Movie S9: Early *P. aeruginosa* localization and effects on a phase-separated SLB containing 1 mol-% Gb3 mix. Representative movie of the early interactions of the *P. aeruginosa* strain PAO1 (GFP-tagged; green) with a phase-separated SLB. Texas Red-DHPE (red) marks the Ld domains. The SLB composition was DOPC/Chol/SM/Gb3 (39.5/20/39.5/1 mol-%) with the supplement of 0.25 mol-% of the fluorescent lipid Texas Red-DHPE. The movie shows the first 15 min after at least 5 bacteria were detectable. The scale bar equals 10 µm.

Movie S10: Early *P. aeruginosa* localization and effects on a phase-separated SLB containing 5 mol-% Gb3 mix. Representative movie of the early interactions of the *P. aeruginosa* strain PAO1 (GFP-tagged; green) with a phase-separated SLB. Texas Red-DHPE (red) marks the Ld domains. The SLB composition was DOPC/Chol/SM/Gb3 (37.5/20/37.5/5 mol-%) with the supplement of 0.25 mol-% of the fluorescent lipid Texas Red-DHPE. The movie shows the first 15 min after at least 5 bacteria were detectable. The scale bar equals 10 µm.

Movie S11: Early *P. aeruginosa* localization and effects on a phase-separated SLB containing 10 mol-% Gb3 mix. Representative movie of the early interactions of the *P. aeruginosa* strain PAO1 (GFP-tagged; green) with a phase-separated SLB. Texas Red-DHPE (red) marks the Ld domains. The SLB composition was DOPC/Chol/SM/Gb3 (35/20/35/10 mol-%) with the supplement of 0.25 mol-% of the fluorescent lipid Texas Red-DHPE. The movie shows the first 15 min after at least 5 bacteria were detectable. The scale bar equals 10 µm.

Movie S12: *P. aeruginosa* binding and effects on a phase-separated SLB containing the synthetic Gb3 analog FSL-Gb3. Representative movie of the interactions of the *P. aeruginosa* strain PAO1 (GFP-tagged; green) with a phase-separated SLB. Texas Red-DHPE (red) marks the Ld domains. The SLB composition was DOPC/Chol/SM/FSL-Gb3 (37.5/20/37.5/5 mol-%) with the supplement of 0.25 mol-% of the fluorescent lipid Texas Red-DHPE. The scale bar equals 10 μm.

Movie S13: Binding and interactions of LecA-coated beads with a phase-separated SLB containing the synthetic Gb3 analog FSL-Gb3. Representative movie of the early interactions of LecA-coated beads (far-red fluorescent; depicted in green) with a phase-separated SLB. Texas Red-DHPE (red) marks the Ld domains. The SLB composition was DOPC/Chol/SM/FSL-Gb3 (37.5/20/37.5/5 mol-%) with the supplement of 0.25 mol-% of the fluorescent lipid Texas Red-DHPE. The movie shows the first 15 min after at least 5 beads were detectable. The scale bar equals 10 μm.

Movie S14: Binding and interactions of LecA-coated beads with a phase-separated SLB containing 0 mol-% Gb3 mix. Representative movie of the early interactions of LecA-coated beads (far-red fluorescent; depicted in green) with a phase-separated SLB. Texas Red-DHPE (red) marks the Ld domains. The SLB composition was DOPC/Chol/SM (40/20/40 mol-%) with the supplement of 0.25 mol-% of the fluorescent lipid Texas Red-DHPE. The movie shows the first 15 min after at least 5 beads were detectable. The scale bar equals 10 μm.

Movie S15: Binding and interactions of LecA-coated beads with a phase-separated SLB containing 1 mol-% Gb3 mix. Representative movie of the early interactions of LecA-coated beads (far-red fluorescent; depicted in green) with a phase-separated SLB. Texas Red-DHPE (red) marks the Ld domains. The SLB composition was DOPC/Chol/SM/Gb3 (39.5/20/39.5/1 mol-%) with the supplement of 0.25 mol-% of the fluorescent lipid Texas Red-DHPE. The movie shows the first 15 min after at least 5 beads were detectable. The scale bar equals 10 μm.

Movie S16: Binding and interactions of LecA-coated beads with a phase-separated SLB containing 5 mol-% Gb3 mix. Representative movie of the early interactions of LecA-coated beads (far-red fluorescent; depicted in green) with a phase-separated SLB. Texas Red-DHPE (red) marks the Ld domains. The SLB composition was DOPC/Chol/SM/Gb3 (37.5/20/37.5/5 mol-%) with the supplement of 0.25 mol-% of the fluorescent lipid Texas Red-DHPE. The movie shows the first 15 min after at least 5 beads were detectable. The scale bar equals 10 μm.

Movie S17: Binding and interactions of LecA-coated beads with a phase-separated SLB containing 10 mol-% Gb3 mix. Representative movie of the early interactions of LecA-coated beads (far-red fluorescent; depicted in green) with a phase-separated SLB. Texas Red-DHPE (red) marks the Ld domains. The SLB composition was DOPC/Chol/SM/Gb3 (35/20/35/10 mol-%) with the supplement of 0.25 mol-% of the fluorescent lipid Texas Red-DHPE. The movie shows the first 15 min after at least 5 beads were detectable. The scale bar equals 10 μm.

Movie S18: Microsphere localization and effects on a phase-separated SLB containing 0 mol-% Gb3 mix. Representative movie of the early interactions of beads (far-red fluorescent; depicted in green) with a phase-separated SLB. Texas Red-DHPE (red) marks the Ld domains. The SLB composition was DOPC/Chol/SM (40/20/40 mol-%) with the supplement of 0.25 mol-% of the fluorescent lipid Texas Red-DHPE. The movie shows the first 15 min after at least 5 beads were detectable. The scale bar equals 10 μm.

Movie S19: Microsphere localization and effects on a phase-separated SLB containing 5 mol-% Gb3 mix. Representative movie of the early interactions of beads (far-red fluorescent; depicted in green) with a phase-separated SLB. Texas Red-DHPE (red) marks the Ld domains. The SLB composition was DOPC/Chol/SM/Gb3 (37.5/20/37.5/5 mol-%) with the supplement of 0.25 mol-% of the fluorescent lipid Texas Red-DHPE. The movie shows the first 15 min after at least 5 beads were detectable. The scale bar equals 10 μm.

Movie S20: Localization of the *P. aeruginosa* LecA knockout mutant strain and effects on a phase-separated SLB containing 0 mol-% Gb3 mix. Representative movie of the interactions of the LecA knockout mutant of the *P. aeruginosa* strain PAO1 (GFP-tagged; green) with a phase-separated SLB. Texas Red-DHPE (red) marks the Ld domains. The SLB composition was DOPC/Chol/SM (40/20/40 mol-%) with the supplement of 0.25 mol-% of the fluorescent lipid Texas Red-DHPE. The scale bar equals 10 μm.

Movie S21: Localization of the *P. aeruginosa* LecA knockout mutant strain and effects on a phase-separated SLB containing 5 mol-% Gb3 mix. Representative movie of the early interactions of the LecA knockout mutant of the *P. aeruginosa* strain PAO1 (GFP-tagged; green) with a phase-separated SLB. Texas Red-DHPE (red) marks the Ld domains. The SLB composition was DOPC/Chol/SM/Gb3 (37.5/20/37.5/5 mol-%) with the supplement of 0.25 mol-% of the fluorescent lipid Texas Red-DHPE. The scale bar equals 10 μm.
